# Effectiveness and safety of Chaihu-Shugan-San for treating depression based on clinical cases: An updated systematic review and meta-analysis

**DOI:** 10.1097/MD.0000000000038668

**Published:** 2024-06-28

**Authors:** Xiaohang Zhang, Qiulong Zhao, Yang Wang, Yaqing Mao, Yue Sun, Xiaokun Bian

**Affiliations:** a Department of Pharmacy, The First People’s Hospital of Yancheng, Yancheng, Jiangsu, China; b School of Integrated Chinese and Western Medicine, Nanjing University of Chinese Medicine, Nanjing, Jiangsu, China; c Department of Pharmacy, Shenzhen Hospital of Integrated Traditional Chinese and Western Medicine, Shenzhen, Guangdong, China; d Department of Pharmacy, Children's Hospital of Soochow University, Suzhou, Jiangsu, China; e School of Pharmacy, Nanjing University of Chinese Medicine, Nanjing, Jiangsu, China.

**Keywords:** antidepressants, Chaihu-Shugan-San, depression, effectiveness, meta-analysis, side effect

## Abstract

**Background:**

Chaihu-Shugan-San (CSS), a Traditional Chinese Medicine formula, has been widely used for treating depression since the Ming Dynasty, as recorded in Jingyue Quanshu, but its effectiveness and safety lack comprehensive and objective evaluation. Based on our meta-analysis, we aimed to adequately evaluate the efficacy and risk of CSS by considering the latest clinical literature.

**Methods:**

Multiple databases, including PubMed, Embase, Web of Science, SinoMed, China National Knowledge Infrastructure, Chongqing VIP, and Wanfang, were used to collect clinical data. The quality of the included clinical studies was assessed using the Cochrane Risk of Bias Tool, and the data were meta-analyzed using Review Manager 5.0 and Stata 17. The data were obtained from a genome-wide association study, and Mendelian randomization (MR) was performed using R Software 4.3.2 with the TwoSampleMR and MR Pleiotropy RESidual Sum and Outlier packages.

**Results:**

A total of 15 studies with 1034 patients and 6 antidepressant drugs were included in this work. Meta-analyses revealed that drug combinations of CSS and antidepressants significantly improved depressive symptoms (weighted mean difference = −4.21; 95% confidence interval [CI]: −5.62–−2.81), increased the effective rate (odds ratio [OR] = 3.82; 95% CI: 2.44–6.83), and reduced side effects (OR = −3.55; 95% CI: −5.66–−1.43) compared with antidepressant monotherapy. Additionally, compared with antidepressant monotherapy, CSS alone exhibited fewer side effects (95% CI:−9.25–−6.95). Like antidepressants, CSS also improved depressive symptoms (weighted mean difference = −0.05; 95% CI: −0.63–−0.52) and increased the effective rate (OR = 1.07; 95% CI: 0.52–2.20). Additionally, MR was used to evaluate the safety of traditional antidepressants, as there was a causal association between amitriptyline and body mass index.

**Conclusion:**

This analysis demonstrated that compared with traditional antidepressants, CSS combined with antidepressants was more effective and safer for treating depressed patients. MR showed that a causal relationship may exist between amitriptyline and body mass index. Therefore, clinicians should carefully consider the advantages and potential drawbacks of Traditional Chinese Medicine and classic drugs to serve patients better.

## 1. Introduction

Depression is a psychiatric disorder characterized by low mood, loss of motivation, feelings of despair, and an inability to feel pleasure.^[1]^ The number of people who suffer from depression worldwide exceeds 300 million (2017 World Health Organization guidelines for Screening for depression, http://t.hk.uy/zYB), which can cause not only mental distress but also induce basic physiological disorders such as those regarding metabolism,^[2]^ autonomic nervous function,^[3]^ functional dyspepsia,^[4]^ and low appetite,^[5]^ leading to suicide or handicaps and a $92.7 billion medical burden annually.

Consequently, numerous “ripe fruits” of antidepressants, such as the classic fluoxetine, escitalopram oxalate, and amitriptyline, have been picked up by large biopharmaceutical companies in the last few decades.^[6]^ Unfortunately, they seem to fail to work^[7,8]^ due to some serious adverse effects, including obesity, cardiovascular dysfunction, sleep disturbances, and gastrointestinal discomfort.^[9,10]^ Given such an awkward situation, Chinese medicines with multitarget effects provide various options^[6]^ for the treatment of depression. According to reports, insomnia^[11]^ and cardiovascular disease^[12]^ could be addressed using Traditional Chinese Medicine (TCM) as a complementary and alternative method. However, concerns about the safety^[13]^ and toxicity^[14,15]^ of TCMs are undeniable.

According to TCM theory, depression is a repressive state of distress and anger.^[16]^ The famous Chinese medicine Chaihu-Shugan-San (CSS) was first recorded in Jingyue Quanshu (Ming Dynasty)^[17]^ and is frequently used in treating liver-Qi stagnation-induced depression in the clinic. Seven herbs, *Citrus reticulata* Blanco, *Bupleurum falcatum* L., *Paeonia lactiflora* Pall., *Cyperus rotundus* L., *Ligusticum striatum* DC., *Citrus* ×* aurantium* L., and *Glycyrrhiza uralensis* Fisch., are prescribed in a ratio of 4: 4: 3: 3: 3: 3: 1.^[18]^ At present, there have been several meta-analyses^[19,20]^ and pharmacodynamic reports^[21–23]^ on CSS; unfortunately, these studies have neglected the safety of CSS due to the absence of updated adverse event-related data. Additionally, studies regarding the risks and effects of interactions between TCM and conventional medication have rarely been reported.^[24]^

Mendelian randomization (MR) is an alternative way to explore the issue of causality in epidemiological research by using genetic variants as the instrumental variable (IV).^[25]^ IVs must meet the following assumptions: they should be closely related to the exposure, they should not be associated with potential confounders of the exposure-outcome association, and they should not influence the outcome by any variable other than the exposure.^[26]^ The above assumptions allow MR to avoid confounders such as lifestyle affecting the judgment of causation between exposure and outcome and better elucidate the results.

Therefore, this review aimed to update the data to support the efficacy and incidence of the major side effects of CSS for patients with depression. Additionally, we used MR to investigate whether a causal relationship exists at the genetic level between classical antidepressants and side effects to provide a further research direction.

## 2. Materials and methods

### 2.1. Meta-analysis

#### 2.1.1. Data sources and search strategy

Searches were conducted for all prospective randomized controlled trials involving CSS treatment in patients suffering from various forms of depression. Seven electronic databases were searched for relevant studies on this topic: PubMed, Embase, Web of Science, SinoMed, China National Knowledge Infrastructure database, Wanfang Database, and Chongqing VIP Information, with the following keywords: “depression,” “depressive disorder,” “depressive-like,” “Chaihu-Shugan-san,” “Chaihu Shugan,” “Chaihu Shugan Powder,” and “Chai Hu Shu Gan.” Peer-reviewed articles published in academic journals from January 1, 2012, to August 31, 2023, were considered. Languages of publication were not restricted in the search.

#### 2.1.2. Criteria for study selection

##### 2.1.2.1. Inclusion criteria

We referred to the inclusion criteria used by Wang et al.^[20]^ Patients were included in the study according to the diagnostic criteria of the Diagnostic and Statistical Manual of Mental Disorders, the International Classification of Diseases, or the Chinese Classification of Mental Disorders, and the level of depressive symptoms was assessed using the Hamilton Depression Rating Scale (HAMD); the study was a randomized controlled trial; CSS (monotherapy or in combination with other antidepressant drugs) was compared with antidepressant drugs; treatment lasted for 2 weeks or more; >30 participants were enrolled.

##### 2.1.2.2. Exclusion criteria

The exclusion criteria were as follows: Ineligible; duplicate publication; lack of access to full-text data; obvious errors in the study design; and secondary depression due to other medical conditions.

#### 2.1.3. Data extraction^[27]^

Two dependent reviewers extracted the included trials’ protocol and treatment outcome data. The information included treatment regimen (monotherapy or combination treatment), controlled conditions (antidepressant drugs), duration of treatment, age, sex ratio, and sample size. We contacted the study authors via e-mail to request missing data or clarification. Two independent reviewers screened titles, abstracts, and full-text articles for eligibility, and a third reviewer resolved disagreements. The *κ* value was used to judge the degree of agreement between the 2 reviewers’ evaluations.^[28]^

#### 2.1.4. Quantitative analysis^[29]^

The Cochrane risk of bias tool was used to assess the quality of the included studies by 2 independent reviewers,^[30]^ and a third reviewer resolved any differences. The quality evaluation ratings are divided into 3 categories: high, low, and unknown.^[31]^

#### 2.1.5. Statistical analyses

The comparison was divided into 2 parts: first, CSS combined with antidepressants compared with antidepressant drugs, and second, CSS compared with antidepressant drugs alone. The data were split into continuous and dichotomous variables reporting weighted mean differences (WMDs) and odds ratios (ORs) with 95% confidence intervals (CIs). Heterogeneity between the results of the trials was tested by the Cochran *Q* and *I*^2^ statistics. If *I*^2^ was <50% and *P* > .1, there was no significant heterogeneity, and a fixed effect model was used^[32]^; otherwise, a random effect model was used for analysis. Meta-regression was used to explore the source of heterogeneity as much as possible, and subgroup analysis was performed for verification. The use of funnel plots and Egger or Begg test were suggested for examining publication bias if the number of trials was near or above 10.^[33]^ As a further step, we performed a trim and fill adjusted analysis to recalculate the ES until the funnel plot was symmetrical.^[34]^ Meta-analyses were performed using RevMan (version 5.0, Cochrane Training) and Stata (version 14.0, Stata Corporation).

The depression symptom-level clinician-rated (HAMD) score was selected as the primary outcome measure. The secondary outcomes included the Treatment Emergent Symptom Scale (TESS), Self-Rating Depression Scale (SDS), and TCM Syndrome Score Scale (TCMSSS) scores.

### 2.2. MR

Among all kinds of traditional antidepressants, amitriptyline has also been frequently used as an active comparator in trials on newer antidepressants since it was first discovered (Elavil: 1961) and could, therefore, be called a “benchmark” antidepressant among many kinds of antidepressants.^[35,36]^ Moreover, obesity is a common side effect of antidepressants,^[37]^ and can be calculated by the body mass index (BMI),^[38]^ accordingly, amitriptyline was selected as the classical antidepressant, and BMI was selected as the typical antidepressant side effect to explore whether there was causality between amitriptyline use and BMI.

#### 2.2.1. Genetic instrument selection

A total of 21 single-nucleotide polymorphisms (SNPs) associated with amitriptyline were identified at the genome-wide significance threshold (*P* < 5 × 10^−6^) in meta-analyses of genome-wide association studies on amitriptyline (about 667,916 individuals). Based on 1000 genomic reference panels limited to European populations, the linkage imbalance between these SNPs per exposure was estimated using the PLINK clustering method.^[39]^ The SNP with the lowest *P* value was retained when linkage disequilibrium (*r*^2^ > 0.001 and clump window = 10,000 kb) was excluded, leaving 3 SNPs as IVs for amitriptyline. More information about instrument selection is presented in Supplementary Table S2, Supplemental Digital Content, http://links.lww.com/MD/M985.

#### 2.2.2. Data source for BMI

Summary-level BMI data were derived from the Biobank study. We used the second wave of MRC-IEU’s genome-wide association studies in the UK Biobank. After excluding individuals of non-European descent, closely related individuals (or at least one of a pair of related individuals), individuals with sex chromosome aneuploidy and amitriptyline information loss, and individuals who dropped out of the UK Biobank study, 461,460 people were recruited.

#### 2.2.3. Statistical analysis

Our statistical analysis methods were performed as described by Yuan et al,^[39]^ and the exposure and outcome data are listed in Table 1. Additionally, the TwoSampleMR (version 0.5.8) and MR Pleiotropy RESidual Sum and Outlier packages (version 1.0) in R Software (version 4.3.2) were used to analyze the data.

**Table 1:** Associations of genetic predisposition to amitriptyline with BMI in MR analyses.

Exposure	Effect estimates on BMI				Test of pleiotropy	
	MR method	OR	95% CI	*P*	Test	
Amitriptyline (21 SNPs)	Inverse variance weighted	4.006	1.611–9.959	.0028	Cochran *Q* value	20
	MR Egger	1.068	0.117–9.749	.9543	MR-Egger intercept (*P*)	0.5711
	Weighted median	2.952	1.469–5.930	.0023	Distortion test (*P*)	0.323

Reverse MR						
	Effect estimates on amitriptyline				Test of pleiotropy	
Exposure	MR method	OR	95% CI	*P*	Test	
BMI (697 SNPs)	Inverse variance weighted	1.009	1.006–1.012	2.44 × 10^−10^	Cochran *Q* value	669
	MR Egger	1.002	0.994–1.011	.5740	MR-Egger intercept (*P*)	0.031
	Weighted median	1.005	1.001–1.010	.0089	Distortion test (*P*)	NA

BMI = body mass index, CI = confidence interval, MR = Mendelian randomization, NA = not available, OR = odds ratio, SNPs = single-nucleotide polymorphisms.

## 3. Results

### 3.1. Meta-analysis

#### 3.1.1. Search results

**Figure 1.**
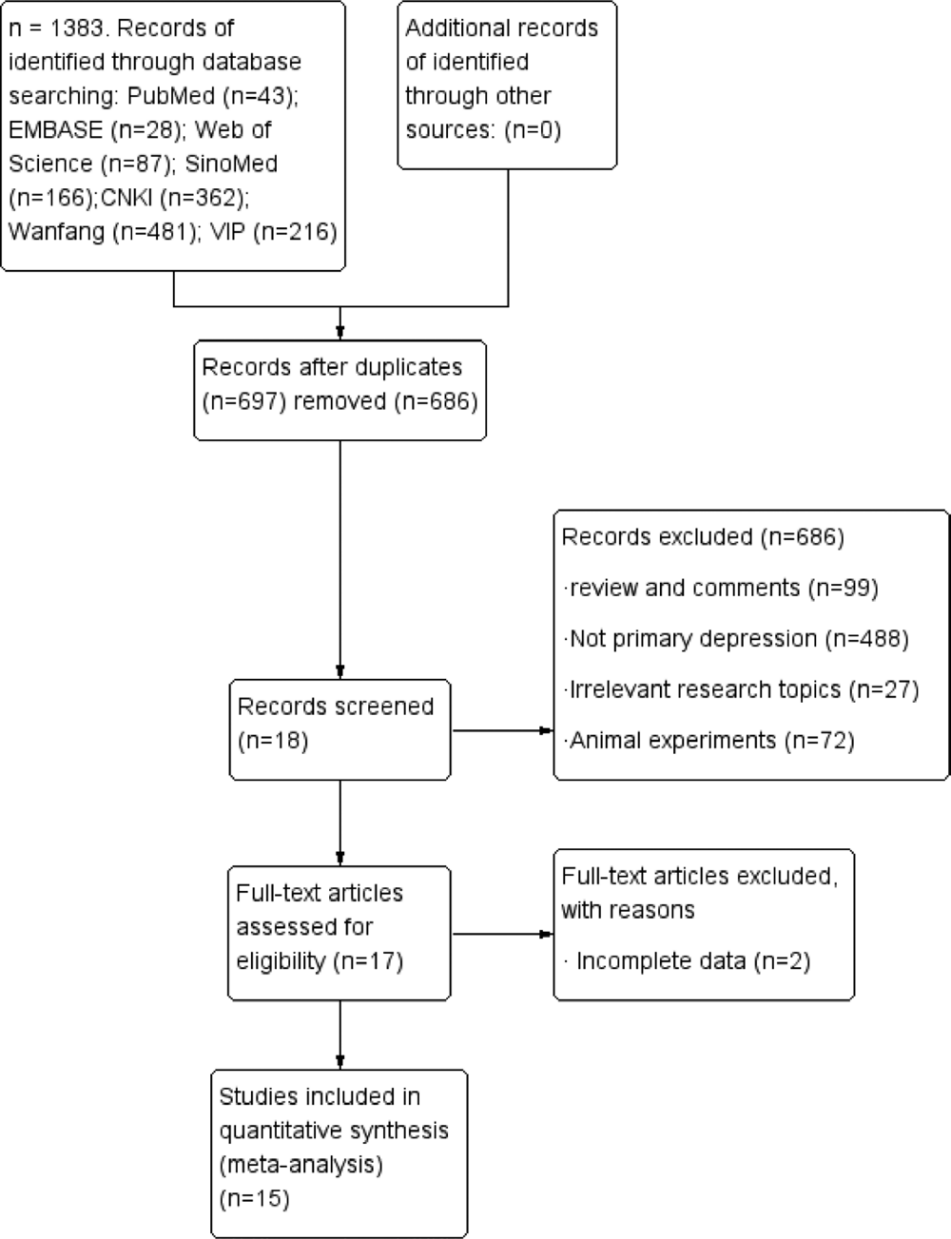
Flow diagram of literature search and selection procedure.

Our literature search identified 1383 publications. After removing duplicates, 697 titles and abstracts were screened. The full texts of 17 articles were read for a detailed evaluation. Overall, 15 studies were included in the meta-analysis. Of these studies, 14^[40–53]^ were in Chinese, and 1^[54]^ was in English (Fig. 1). The consistency of the evaluation passed the *κ* test (*κ* = 0.827). This work was registered with PROSPERO: CRD42024543977.

#### 3.1.2. Characteristics of the included studies

A total of 15 studies^[40–54]^ were analyzed in this study. The included studies enrolled 1027 patients, divided into an observation group (n = 501) and a control group (n = 495). Five of the studies^[41–43,50,51]^ used paroxetine in the control group. In 2 studies,^[44,47]^ venlafaxine was used as the control. In 3 studies,^[52–54]^ fluoxetine was used as the control, and in 1 study,^[45]^ amitriptyline was used as the positive control drug. The trial duration ranged from 2 weeks to 3 months. All patients received oral interventions.

There were 2 studies^[52,53]^ without detailed sex information, 3 studies^[41,43,52]^ without detailed age information, and 6 studies^[40,41,43,44,47,54]^ that did not report the disease duration. Fifteen studies examined HAMD,^[40–54]^ 6 studies evaluated TESS,^[40,43,46,47,49,51]^ 3 studies examined TCMSSS^[50,52,53]^ and SDS,^[48,50,53]^ and 10 studies reported the treatment effect (effective rate).^[43–45,47,49–53]^ Detailed information about the treatment of patients with depression and CSS in each study is displayed in Table 2.

**Table 2:** Basic characteristics of the included studies.

Author	n		Sex (men (n, %))		Age		Intervention		Dose	Course		Treatment duration (wk)	Outcomes
	T	C	T	C	T	C	T	C		T	C		
Qiu et al 2014	20	20	8, 40	6, 31	30.6 ± 8.9	30.1 ± 9.0	CSS	FLX	FLX: 20–40 mg; CSS: 300 mL twice daily	Unknown	Unknown	8	HAMD
Li et al 2022	30	30	Unknown	Unknown	38.77 ±3.64	38.65 ±3.56	CSS + FLX	FLX + PBO	FLX: 20 mg/d orally after breakfast. After 1 wk, 40 mg/d; CSS: 150 mL/d; PBO: 150 mL/d	4.45 ± 0.77 yr	4.39 ± 0.70 yr	8	HAMD; effective rate; TCMSSS; SDS
Gu 2016	30	30	18, 60	16, 53.33	33.1 ± 14.4	32.8 ± 17.7	CSS + MIR	MIR	MIR: 15 mg/d, max: 45mg/d; CSS: 1 dose/d, 2 times	16.6 ± 10.8 wk	16.1 ± 12.5 wk	9	HAMD; effective rate; TESS
Xu et al 2016	40	40	14, 35	15, 37.5	41.02 ±4.89	40.34 ±5.15	CSS	PX	PX: 20 mg/d; CSS: 200 mL/d	2 ± 0.54 mo	2.24 ± 0.63 mo	2	HAMD; TESS
Liu et al 2016	50	50	23, 46	24, 48	51.61 ±4.03	51.29 ±7.15	CSS	PX	PX: 20 mg/d; CSS: 200 mL/d	8 ± 1.46 mo	8.98 ± 1.47 mo	4	HAMD
Liu et al 2014	30	30	27	45	48.3 ± 35.77		CSS	PX	PX: 20mg/d; CSS: 200 mL/d	8.83 ± 4.72 mo		4	HAMD
Liu et al 2012	30	32	11, 36.67	12, 37.5	40.5 ± 9.9	36.6 ± 15	CSS + VLF	VLF	VLF: 75 mg/d, after 1 wk, 150 mg/d; CSS: 300 mL/d	Unknown	Unknown	4	HAMD; effective rate; TESS
Li et al 2021	30	30	Unknown	Unknown	19–56	21–60	CSS + FLX	FLX + PBO	FLX: 20 mg/d orally after breakfast. After 1 wk, 40 mg/ d; CSS: 150 mL/d; PBO: 150 mL/d	1.5–7 yr	2–8 yr	8	HAMD; effective rate; TCMSSS
Yang 2019	29	28	10, 34.48	10, 35.71	46.9 ± 9.244	47.7 ± 7.637	CSS + PX	PX	PX: 20 mg/d; CSS: 400 mL/d	6.38 ± 1.613 mo	6.5 ± 1.291 mo	8	HAMD; effective rate; TCMSSS; SDS
Sun 2020	48	48	21, 43.75	22, 45.83	39.86 ± 7.74	39.63 ±7.51	CSS + ESO	ESO	ESO: 20 mg/d; CSS: 300 mL/d	3.61 ± 1.12 yr	3.65 ± 1.14 mo	8	HAMD; SDS
Huang 2018	24	23	13, 54.17	12, 52.13	48.1 ± 3.2	48.5 ± 3.1	CSS + AMI	AMI	AMI: 25 mg/d, after 3 d, 50 mg/d; CSS: 350 mL/d	4.2 ± 0.6 mo	4.5 ± 0.7 mo	12	HAMD; effective rate
Hu et al 2017	40	40	22, 55	24, 60	39.52 ± 1.25	38.45 ±1.27	CSS + CIL	CIL	CIL: 20 mg/d, 2 times/d, after 1 wk, 40 mg/d; CSS: 1 dose/d	Unknown	Unknown	4	HAMD; TESS
Wang and Zhu 2013	40	40	18, 45	21, 52.5	33.6 ± 10.75	34.7± 11.23	CSS	PX	PX: 20 mg/d, after 1 wk, 20–30 mg/d; CSS: 200 mL/d	11.6 ± 9.7 mo	12.1 ± 8.3 mo	6	HAMD; effective rate; TESS
Liu et al 2012-1	20	21	7, 35	6, 28.57	45.5 ± 13.3	36.8 ± 15.7	CSS + VLF	VLF	VLF: 75 mg/d, after 1 wk, 150 mg/d; CSS: 300 mL/d	Unknown	Unknown	4	HAMD; effective rate

AMI = amitriptyline, C = control group, CIL = citalopram, CSS = Chaihu-Shugan-San, ESO = escitalopram oxalate, FLX = fluoxetine, HAMD = Hamilton Rating Scale for Depression; effective rate, MIR = mirtazapine, PBO = placebo, PX = paroxetine hydrochloride, SDS = Self-Rating Depression Scale, T = treatment group, TCMSSS = Traditional Chinese Medicine Syndrome Score Scale, TESS = Treatment Emergent Symptom Scale, VLF = venlafaxine.

#### 3.1.3. Quantitative analysis

Our quality assessment is illustrated in Supplementary Figure S1, Supplemental Digital Content, http://links.lww.com/MD/M988. Overall, the quality of all articles was low risk, and few trials reported adequate information on outcome assessor blinding and cointervention.

#### 3.1.4. Meta-analytic results

##### 3.1.4.1. The primary outcomes of CSS combined with antidepressant drugs versus antidepressant drugs alone

**Figure 2.**
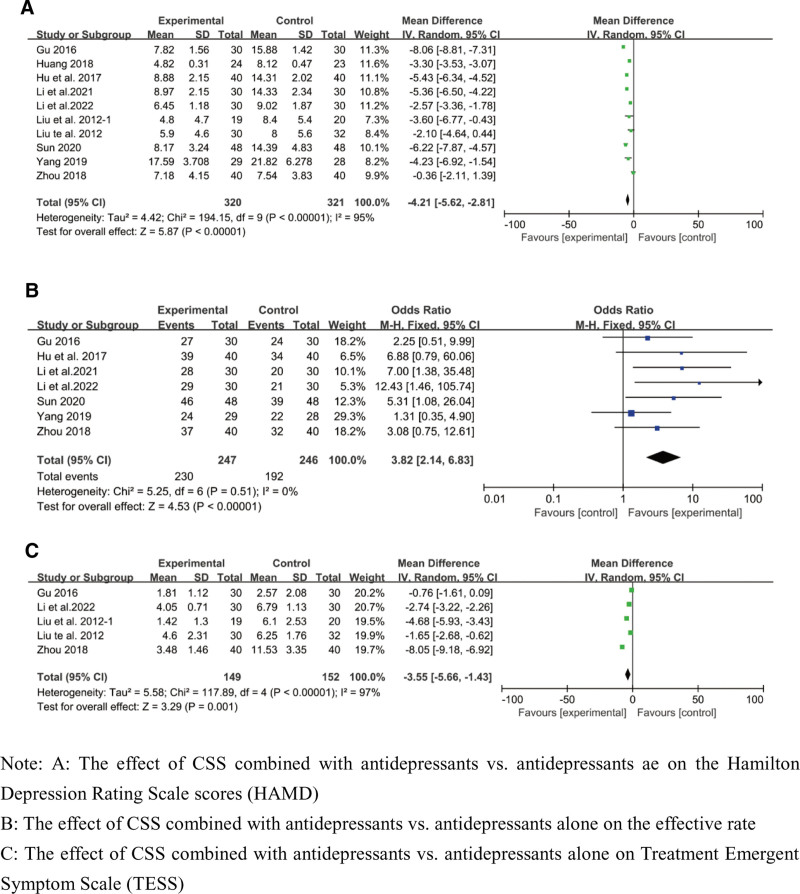
Main outcomes: Chaihu-Shugan-San combined with antidepressants versus antidepressants alone.

As the primary outcome, ten studies, including 641 patients, contributed to the HAMD.^[40,43–45,47–50,52,53]^ The data were analyzed using a random-effects model, which revealed significant heterogeneity (*P* < .00001; *I*^2^ = 95%) and a significant difference between the above 2 groups (320 in the treatment group and 321 in the control group), with an SMD of − 4.21 (95% CI: −5.62–−2.81; *P* < .00001) (Fig. 2A). Seven studies^[40,43,48–50,52,53]^ involving an overall sample of 493 patients (247 in the treatment group and 249 in the control group) were conducted for effective rate and reported significant antidepressant effects according to a fixed-effects model (*P* = .51; *I*^2^ = 0%), with an OR of 3.82 (95% CI: 2.14–6.83; *P* < .0001) (Fig. 2B). Five studies contributed to TESS,^[43,44,47,49,53]^ resulting in an overall sample of 301 patients. The data were analyzed using a random-effects model (*P* < .00001; *I*^2^ = 97%). According to the included trials, the core of TESS in the studies of patients who received CSS treatment (n = 149) was less than that in the studies of patients who did not receive CSS treatment (n = 152), with an OR of −3.55 (95% CI: −5.66–−1.43; *P* = .001) (Fig. 2C).

#### 3.1.5. The *primary* outcomes of CSS versus antidepressant drugs

Five studies contributed to the HAMD,^[41,42,46,51,54]^ with an overall sample of 353 patients and no heterogeneity (*P* = .81; *I*^2^ = 0%). After merging the results, the WMD for CSS combined with antidepressant drugs (n = 180) versus antidepressant drugs alone (n = 16) was −0.05 (95% CI: −0.63–−0.52; *P* = .85), showing no significant difference in the HAMD score (Supplementary Fig. S2A, Supplemental Digital Content, http://links.lww.com/MD/M989), which is in line with the findings of 3 studies. Using a fixed-effects model, 2 studies,^[46,51]^ including 160 patients, contributed to the effective rate (*P* = .81; *I*^2^ = 0%). The results revealed no significant difference between the CSS treatment group (n = 80) and the no-CSS treatment group (n = 80), and the OR was 1.07 (95% CI: 0.52–2.20; *P* = .85) (Supplementary Fig. S2B, Supplemental Digital Content, http://links.lww.com/MD/M989), which was also similar to the findings of 2 other studies. Only 1 study,^[51]^ with 80 patients (half in both groups), reported TESS, with an OR that was −8.1 lower than that for patients receiving antidepressant treatment (95% CI: −9.25–−6.95; *P* < .0001).

#### 3.1.6. Other outcomes

The treatments used for the experimental group and the control group were CSS combined with antidepressants and antidepressants alone, respectively.

Three studies,^[48,50,53]^ including 213 patients, reported SDS scores. Patients receiving treatment (n = 107) had lower core SDS during the treatment period than patients receiving antidepressant treatment (n = 106), with an OR of −1.64 (95% CI: −2.76–−0.52; *P* = .004) (Supplementary Fig. S3A, Supplemental Digital Content, http://links.lww.com/MD/M990).

Three studies^[50,52,53]^ involving 177 patients reported TCMSSS scores. Over the treatment period, the core TCMSSS score in patients receiving combination treatment (n = 89) was lower than that in patients receiving antidepressant monotherapy (n = 88), with an OR of −4.00 (95% CI: −5.77–−2.23; *P* < .00001) (Supplementary Fig. S3B, Supplemental Digital Content, http://links.lww.com/MD/M990).

#### 3.1.7. Meta-regression

Meta-regression analyses were employed to explain the observed heterogeneity. The year of publication, type of antidepressant, and length of intervention were chosen as the factors affecting the clinical efficacy of antidepressants for the meta-regression analysis. However, the results showed that the year of publication (*P* = .137) and type of antidepressant (*P* = .276) were not significantly related to heterogeneity, while the length of intervention (*P* = .042) may have led to heterogeneity (Supplementary Table S1, Supplemental Digital Content, http://links.lww.com/MD/M984). Subgroup analysis was subsequently performed to verify this possibility; the results ruled out the possibility (Supplementary Fig. S4, Supplemental Digital Content, http://links.lww.com/MD/M991).

#### 3.1.8. Publication bias

Funnel plot analyses were used to visually detect publication bias. The funnel plot for the HAMD (Supplementary Fig. S6, Supplemental Digital Content, http://links.lww.com/MD/M993) of CSS combined with antidepressants showed no symmetry, the *P* value for Egger test of publication bias on the HAMD was 0.002 (*P* < .05), indicating that publication bias existed in this meta-analysis. To confirm the reliability of our results, the clipping and filling method was used to modify the results, with no reversal, proving that the results have a certain stability.

Fortunately, the funnel plots for TESS (Supplementary Fig. S7, Supplemental Digital Content, http://links.lww.com/MD/M994) and the HAMD (Supplementary Fig. S8, Supplemental Digital Content, http://links.lww.com/MD/M995) of the CSS group showed bilateral symmetry, with *P* values for Egger test of publication bias of 0.081 (*P* > .05) and 0.877 (*P* > .05), respectively.

#### 3.1.9. Sensitivity analysis

Due to high heterogeneity, we sequentially omitted each study to analyze the sensitivity of the results (Supplementary Fig. S5, Supplemental Digital Content, http://links.lww.com/MD/M992), which showed stable results (Es = −2.18, 95% CI: −3.06*–*−1.30).

### 3.2. MR

**Figure 3.**
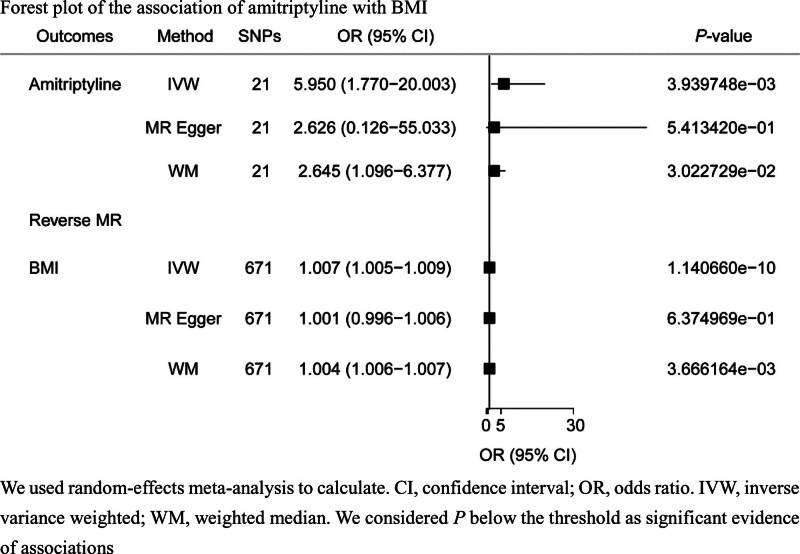
Forest plot of the association of amitriptyline with BMI. BMI = body mass index.

The characteristics of the SNPs used in this study are presented in Supplementary Table S3, Supplemental Digital Content, http://links.lww.com/MD/M986. There were 21 and 614 SNPs as IVs for amitriptyline and BMI, respectively. The positive association between amitriptyline and BMI was replicated in the dataset. The OR for the effect of amitriptyline on BMI was 5.95 (95% CI: 1.77–20.00, *P* = .0039). The sensitivity analysis showed stable results (Fig. 3, Supplementary Table S4, Supplemental Digital Content, http://links.lww.com/MD/M987). Heterogeneity was observed in this analysis, while the MR Pleiotropy RESidual Sum and Outlier global test indicated the absence of pleiotropic effects of amitriptyline. Moreover, scatter, forest, and funnel plots are presented in Supplementary Figs. S9–S16, Supplemental Digital Content, http://links.lww.com/MD/M996, http://links.lww.com/MD/M997, http://links.lww.com/MD/M998, http://links.lww.com/MD/M999, http://links.lww.com/MD/M1000, http://links.lww.com/MD/N2, http://links.lww.com/MD/N3, http://links.lww.com/MD/N4.

Reverse MR suggested that the effect of BMI is also likely to increase the amitriptyline dosage (Supplementary Table S4, Supplemental Digital Content, http://links.lww.com/MD/M987).

## 4. Discussion

Depression is one of the most prevalent public health conditions worldwide.^[55]^ Antidepressants are almost ineffective in 30% to 40% of patients with depression^[56]^ and cause many side effects, while CSS was reported to be effective, well-tolerated, and safe for treating depression.^[54]^ Although a positive influence on depression was detected in animals^[19]^ and clinical trials,^[20]^ the results might be unrealistic because of limitations in methodology, imprecise reporting, and incomplete data (incidence of adverse reactions). Therefore, the efficacy and safety of CSS treatments for depression still need to be supported by more clinical studies.^[57,58]^

CSS monotherapy was superior to antidepressants alone for improving side effects in 5 studies involving 353 depressive patients (95% CI: −9.25–−6.95; *P* < .0001). Moreover, no statistically significant differences in depressive symptoms (WMD = −0.05; 95% CI: −0.63–−0.52) or the effective rate (OR = 1.07; 95% CI: 0.52–2.20) were found between the 2 groups. CSS appears to work similarly to antidepressants in reducing depression, while CSS reduces side effects more effectively than antidepressants. Ten of the studies involving 641 depressive patients evaluated CSS combined with antidepressants compared to antidepressants alone. CSS treatment better improved depressive symptoms (WMD = −4.21; 95% CI: −5.62–−2.81), the effective rate (OR = 3.82; 95% CI: 2.44–6.83), and ameliorated side effects (OR = −3.55; 95% CI: −5.66–−1.43). Based on these results, CSS combined with antidepressants was deemed quite effective at attenuating depression.

Heterogeneity was observed (*P* < .05, *I*^2^ > 70%) in the HAMD and TESS analyses (Figs. 2A and 3B), primarily due to the quality of the CSS formula. In each trial, CSS was used in a different way, which made assessing its effectiveness difficult. Therefore, the standardization of CSS should be addressed. Heterogeneity might also have resulted from the length of intervention between trials. Moreover, the diagnostic criteria, education degree, and age might also have contributed to the heterogeneity. Simultaneously, the high heterogeneity could have led to publication bias, reducing the study’s credibility.

This is the first MR investigation to assess the associations of side effects with antidepressants from a genetic perspective, and in this study, amitriptyline was used as a classical antidepressant. BMI was also a typical antidepressant side effect. Genetic correlation analysis revealed that BMI was strongly correlated with amitriptyline in gene aspects (OR = 5.95, 95% CI: 1.77–20.00; *P* = .0039). Additionally, reverse MR showed the same results, suggesting that classical antidepressants may alter some of the body’s genes to cause severe side effects, resulting in severe harm. However, there is no gene database related to TCM, which needs to be addressed to better calculate the safety and effectiveness of TCM.

There are 2 aspects to the safety of Chinese medicine. Most TCM substances are derived from herbal plants, animals, and minerals.^[59]^ The other is the principle of “treatment based on syndrome differentiation,” and “overall adjustment” of TCM can avoid side effects^[60]^ so that TCM can be safer and more effective in the treatment of depression. Modified CSS, a TCM compound, follows a dynamic prescription (CSS) that adjusts according to the specific condition of the disease. It has been extensively utilized in the treatment of depression and has demonstrated remarkable efficacy and minimal adverse effects. One review revealed that the antidepressive effects of TCM are related to 6 mechanisms.^[61]^ Ma et al^[18]^ demonstrated that CSS can alter the gut microbiota and the levels of bile acids, hyocholic acid, and 7-ketoDCA to alleviate depression-like behavior in mice. Other studies^[62,63]^ have demonstrated that CSS can regulate hippocampal neurons and neurotrophic factors and immune cytokines, increase monoamine neurotransmitter levels (DA, 5-HT), and inhibit hyperactivity of the hypothalamic-pituitary-adrenal axis.

There are several limitations in the study. First, we found that the number of studies was small, as was the sample size, which may have contributed to the high publication bias. Additionally, the trials conducted so far have been of relatively low quality. It is necessary to perform more large, randomized studies with reliable designs to assess the clinical benefits, long-term effectiveness, and incidence of adverse effects of CSS in treating depression. We suggest that future research follow up with patients to obtain long-term clinical data and data on the incidence of adverse events to better explain the effectiveness and safety of traditional antidepressants. Second, the studies included in the analysis were published in English and Chinese, and all studies were conducted in Asian countries. Therefore, more studies should be conducted in various countries to determine whether CSS can benefit every patient regardless of where they come from. Finally, only 1 traditional antidepressant and side effect was employed for MR, and there are many other kinds of antidepressants; consequently, more MR studies are urgently needed to explore the causal relationship between other antidepressants and common side effects.

For these reasons, each subdiagnosis group should receive a standard treatment to achieve higher homogeneity and less publication bias.

## 5. Conclusion

In conclusion, this meta-analysis showed that CSS alone or combined with antidepressants can effectively minimize the side effects of antidepressants, which provides a paradigm for appraising the effectiveness and safety of TCM. Our findings should, however, be interpreted cautiously since they still depend on a small number of trials and subjects. Therefore, future research should address this deficit to help us better understand the mechanisms underlying the efficacy and safety of CSS in depressed patients.

## Acknowledgments

We are extremely grateful to the researchers and providers who have made the public data available for this study, as well as to all the participants who have taken part in this research.

## Author contributions

**Conceptualization:** Xiaohang Zhang, Yue Sun.

**Data curation:** Yue Sun.

**Formal analysis:** Yaqing Mao.

**Funding acquisition:** Yang Wang, Xiaokun Bian.

**Investigation:** Yang Wang, Xiaokun Bian.

**Methodology:** Xiaohang Zhang, Yang Wang, Yaqing Mao.

**Software:** Xiaohang Zhang.

**Supervision:** Xiaokun Bian.

**Visualization:** Yang Wang, Xiaokun Bian.

**Writing – original draft:** Xiaohang Zhang, Qiulong Zhao.

**Writing – review & editing:** Xiaohang Zhang, Qiulong Zhao.

## Supplementary Material








































