# Supplementary material for: Effectiveness and safety of Chaihu-Shugan-San for treating depression based on clinical cases: An updated systematic review and meta-analysis
Source: Medicine (Baltimore). 2024 Jun 28;103(26):e38668. doi: 10.1097/MD.0000000000038668 (PMC11466128; doi:10.1097/MD.0000000000038668)
Supplement: Supplementary file 1 [file medi-103-e38668-s001.docx]

| **Table S2. Information on genetic instruments and outcome data sources.** | | | | | |
| --- | --- | --- | --- | --- | --- |
| Exposure | Unit | Participants included in analysis | Identified SNPs | Used instruments | PubMed ID or Web link |
| Amitriptyline | SD | 667,915 European descent individuals | 21 | 21 | UK Biobank (http:// www.nealelab.is/ uk-biobank) |
|  |  |  |  |  |  |
| BMI | SD | 461,460 European descent individuals | 700 | 697 | UK Biobank (http:// www.nealelab.is/ uk-biobank) |
| Note: PubMed ID: PubMed identifier; SD: standard deviation; SNPs: single-nucleotide polymorphisms; BMI: body mass index. | | | | | |
