# Supplementary figures and images for: Effectiveness and safety of Chaihu-Shugan-San for treating depression based on clinical cases: An updated systematic review and meta-analysis
Source: Medicine (Baltimore). 2024 Jun 28;103(26):e38668. doi: 10.1097/MD.0000000000038668 (PMC11466128; doi:10.1097/MD.0000000000038668)

**
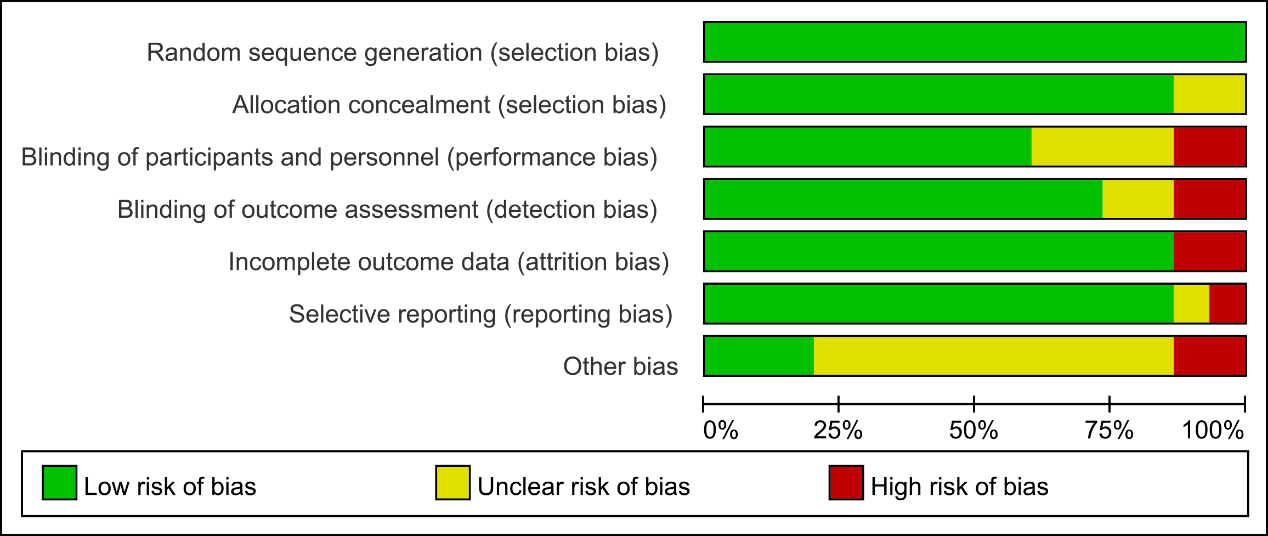
**

**Fig. S1 The risk of bias assessment of included studies.**

Supplement: Supplementary file 2 [file medi-103-e38668-s002.docx]

**
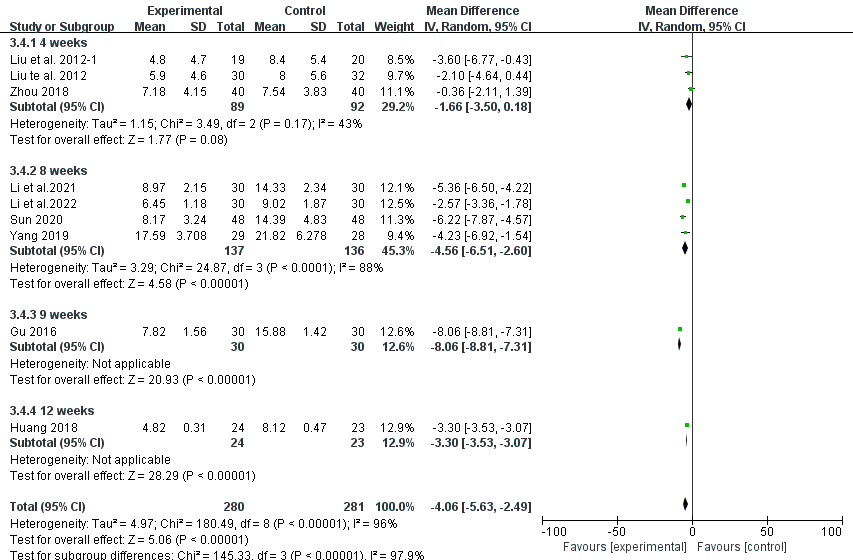
**

**Fig. S4 Subgroup analysis was performed according to course of treatment (HAMD)**

Supplement: Supplementary file 6 [file medi-103-e38668-s006.docx]

**
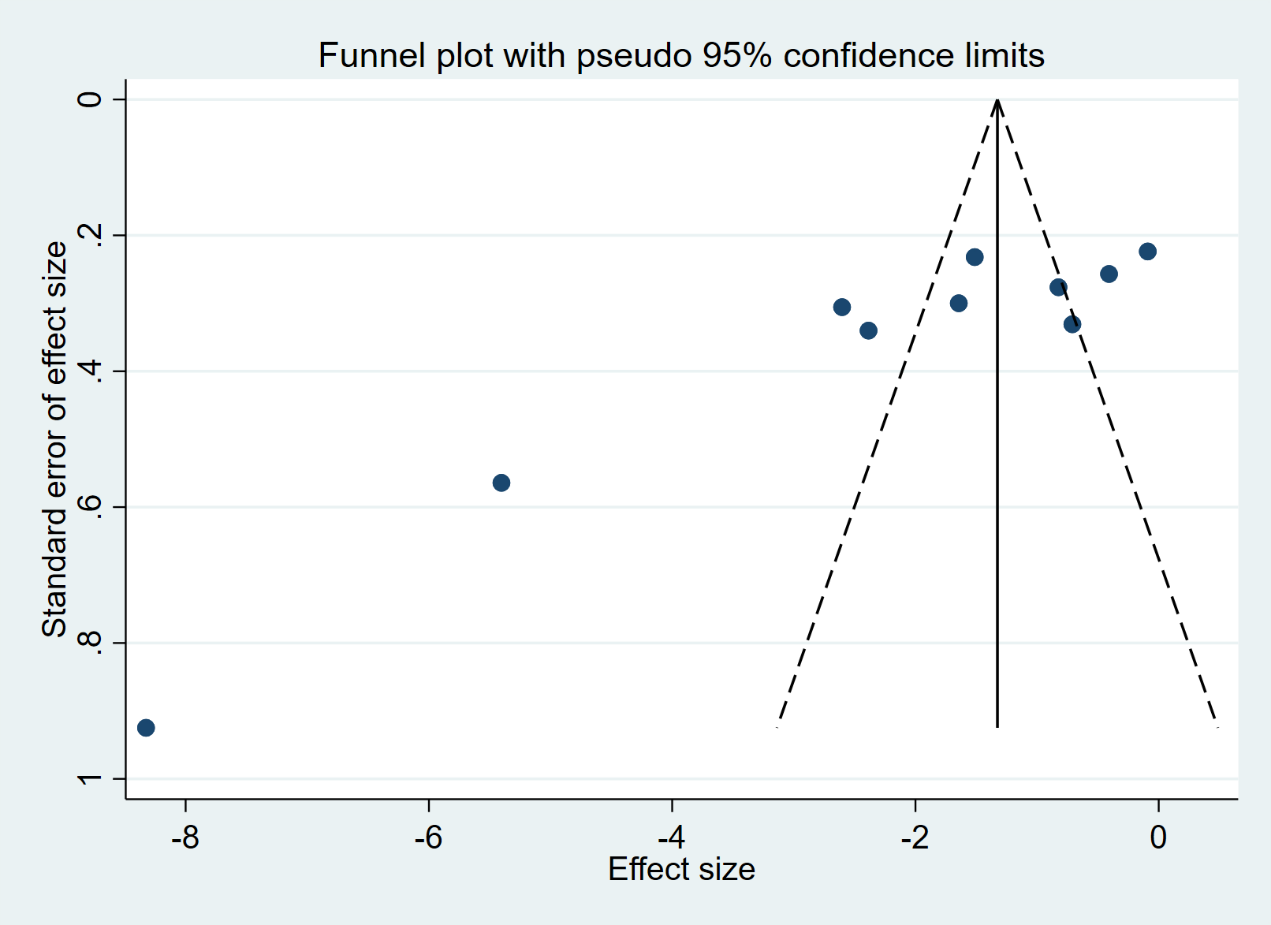
**

**Fig. S6** Funnel plot of the HAMD of CSS combined with antidepressants.

Supplement: Supplementary file 7 [file medi-103-e38668-s007.docx]

**
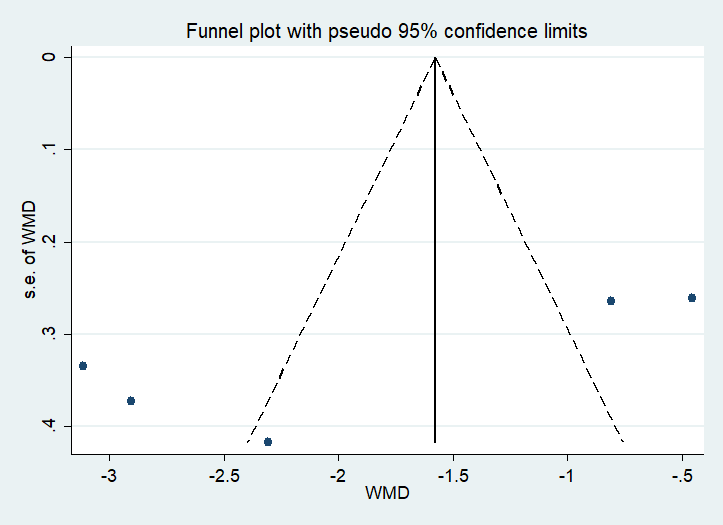
**

**Fig. S7** Funnel plot of the TESS of CSS combined with antidepressants.

Supplement: Supplementary file 8 [file medi-103-e38668-s008.docx]

**
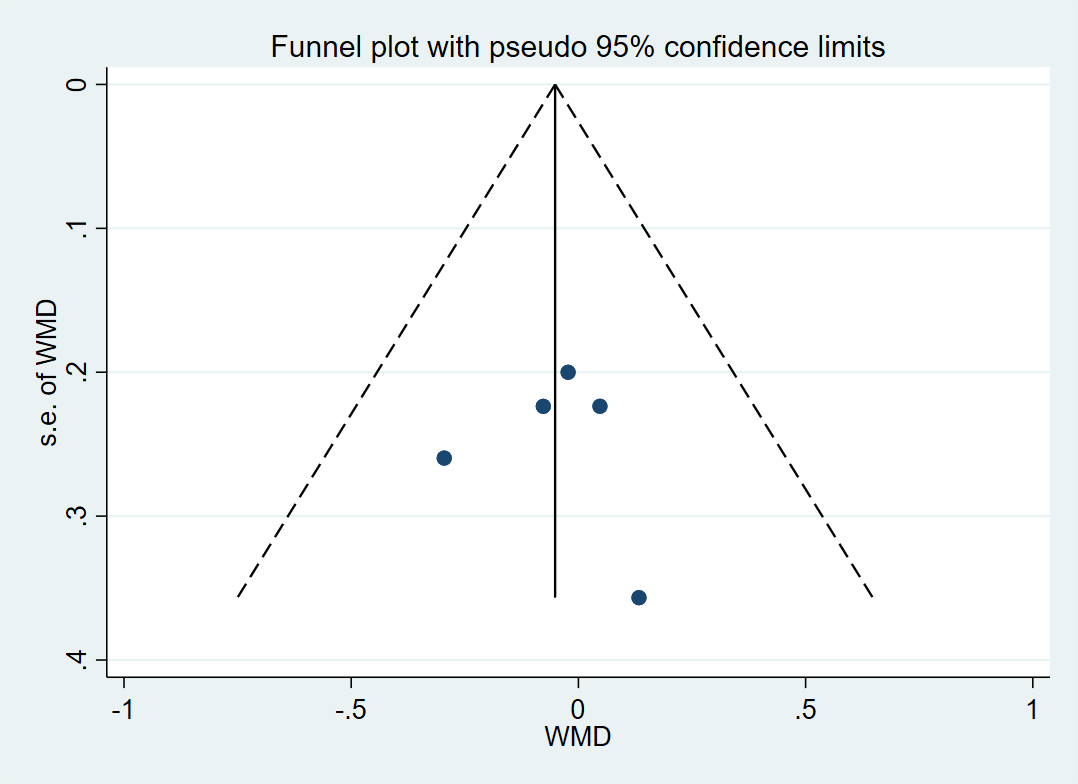
**

**Fig. S8** Funnel plot of the HAMD of CSS.

Supplement: Supplementary file 9 [file medi-103-e38668-s009.docx]

**
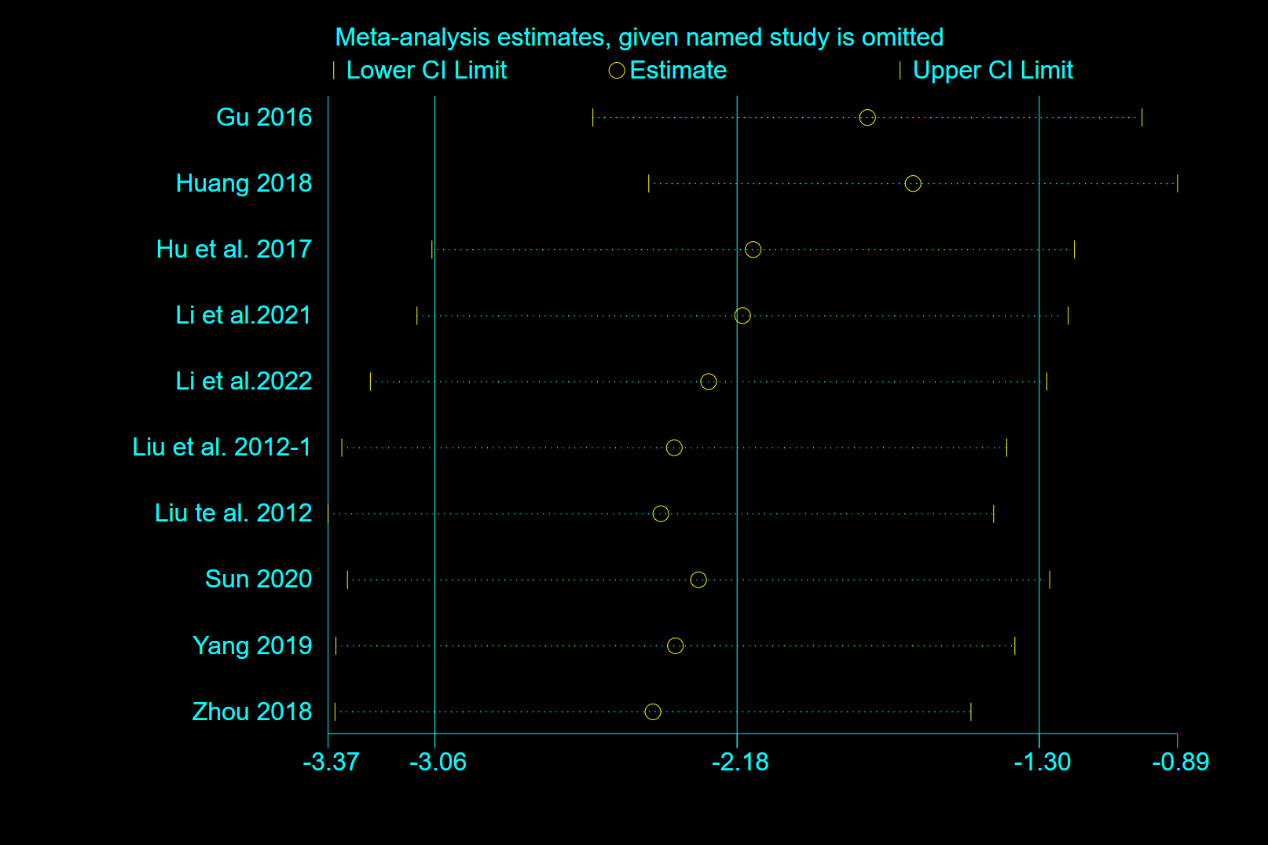
**

**Fig. S5 Sensitivity analysis based on the HAMD of CSS combined with antidepressants.**

Supplement: Supplementary file 10 [file medi-103-e38668-s010.docx]

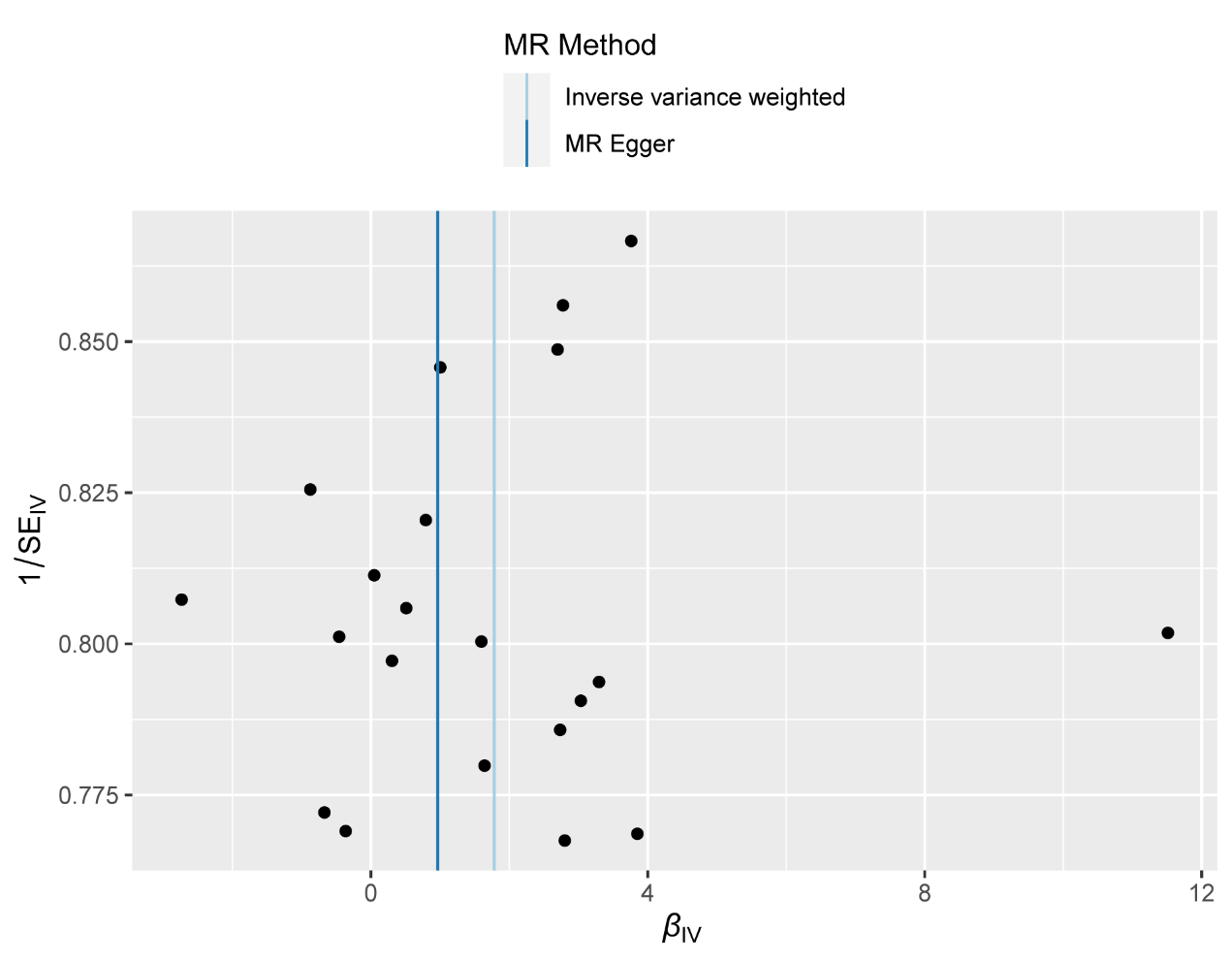


**Fig.S9 the funnel plot of the effect of amitriptyline on body mass index**

Supplement: Supplementary file 13 [file medi-103-e38668-s013.docx]

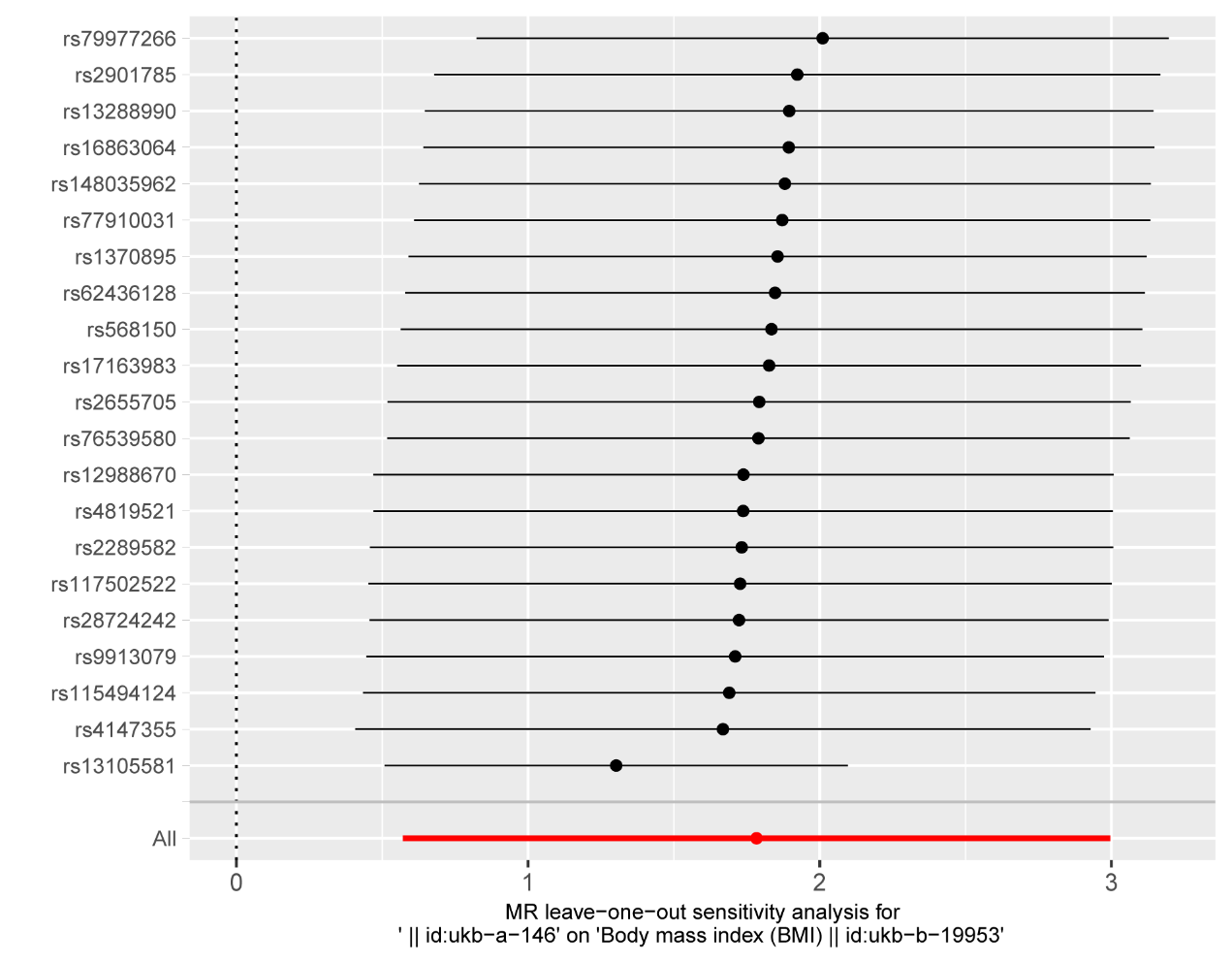


**Fig.S10 the leave-one-out plot of the effect of amitriptyline on body mass index**

Supplement: Supplementary file 14 [file medi-103-e38668-s014.docx]

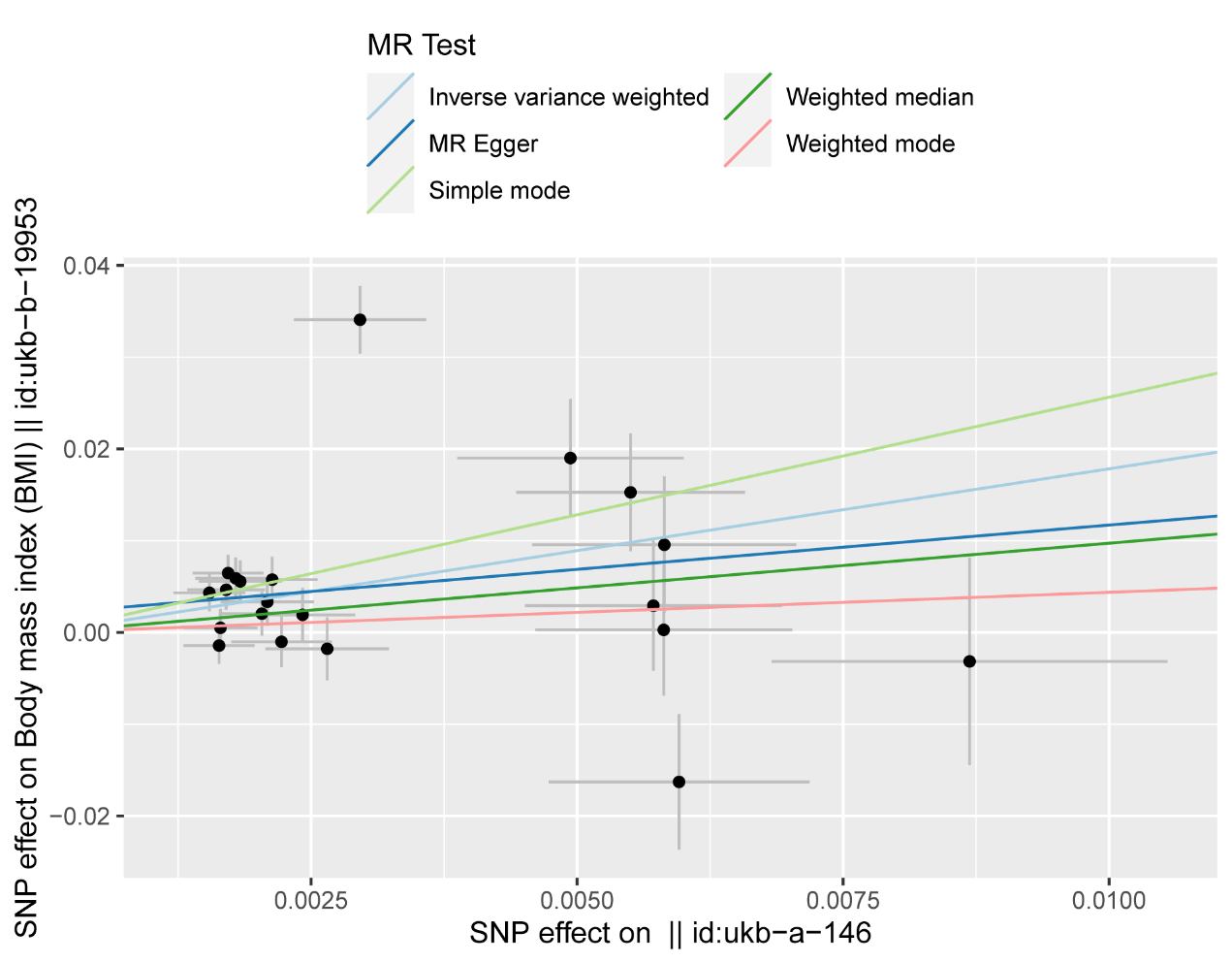


**Fig.S11 the scatter plot of the effect of** **amitriptyline on body mass index**

Supplement: Supplementary file 15 [file medi-103-e38668-s015.docx]

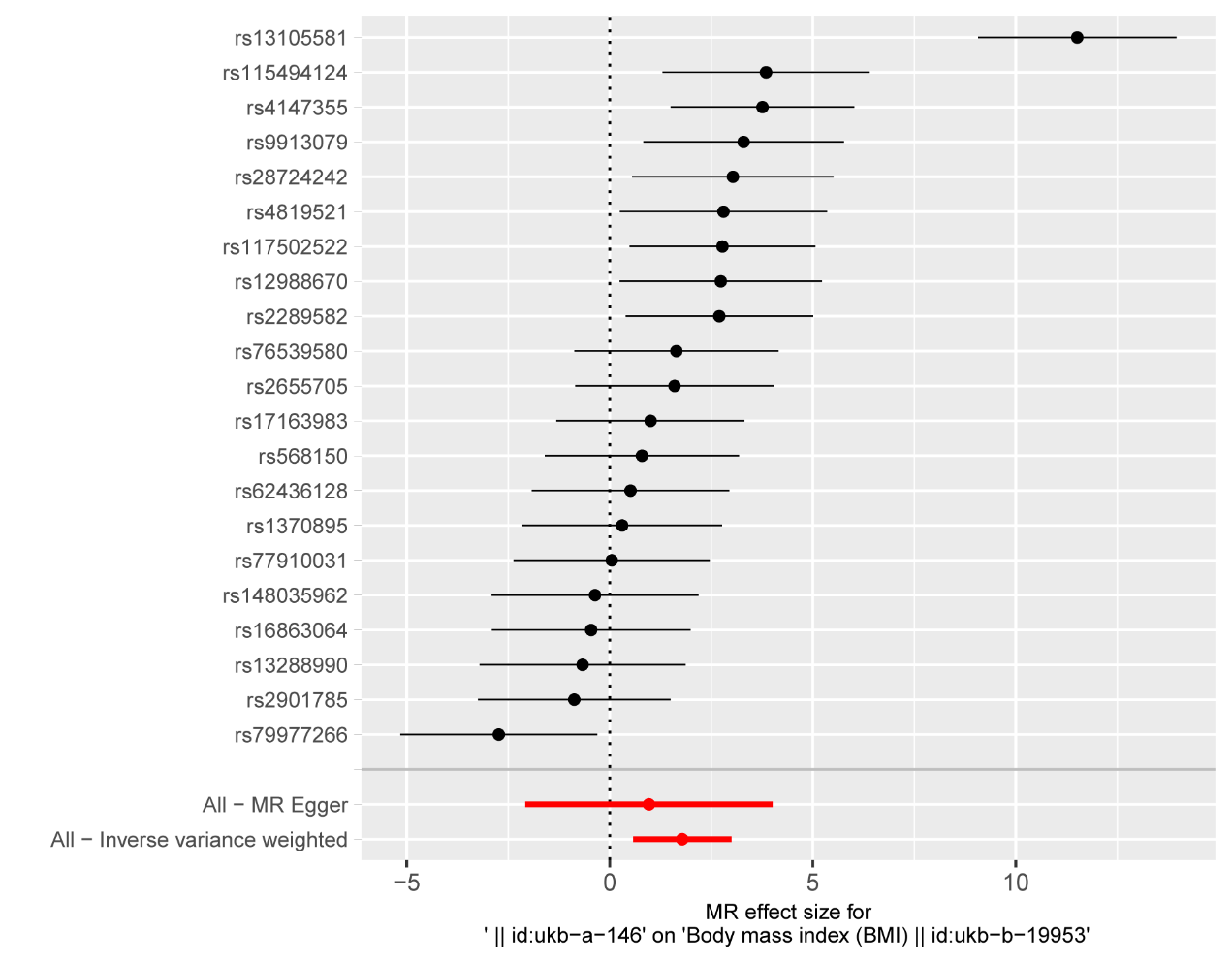


**Fig.S12 the forest plot of the effect of amitriptyline on body mass index**

Supplement: Supplementary file 16 [file medi-103-e38668-s016.docx]

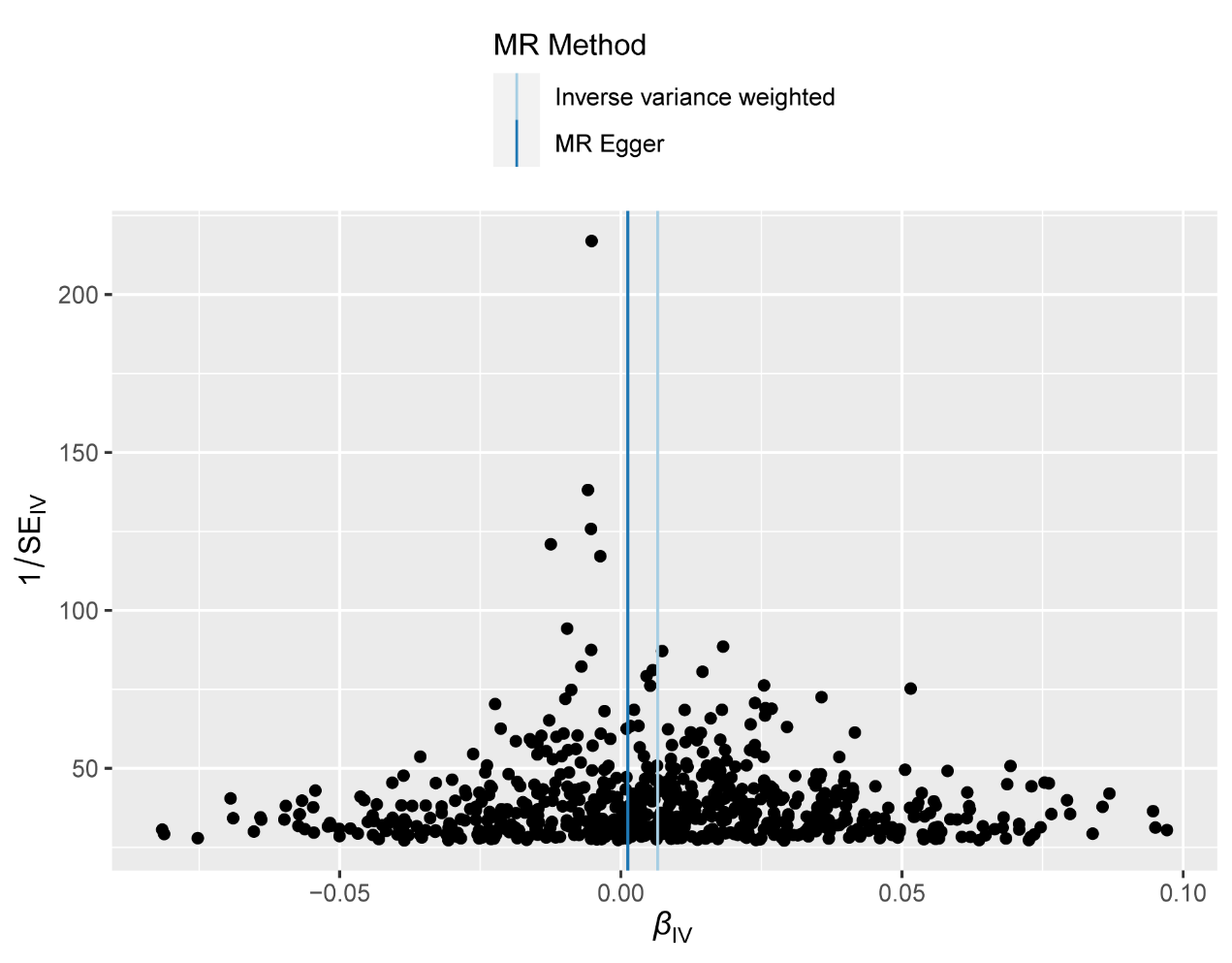


**Fig.S13 the funnel plot of the effect of body mass index on amitriptyline**

Supplement: Supplementary file 17 [file medi-103-e38668-s017.docx]

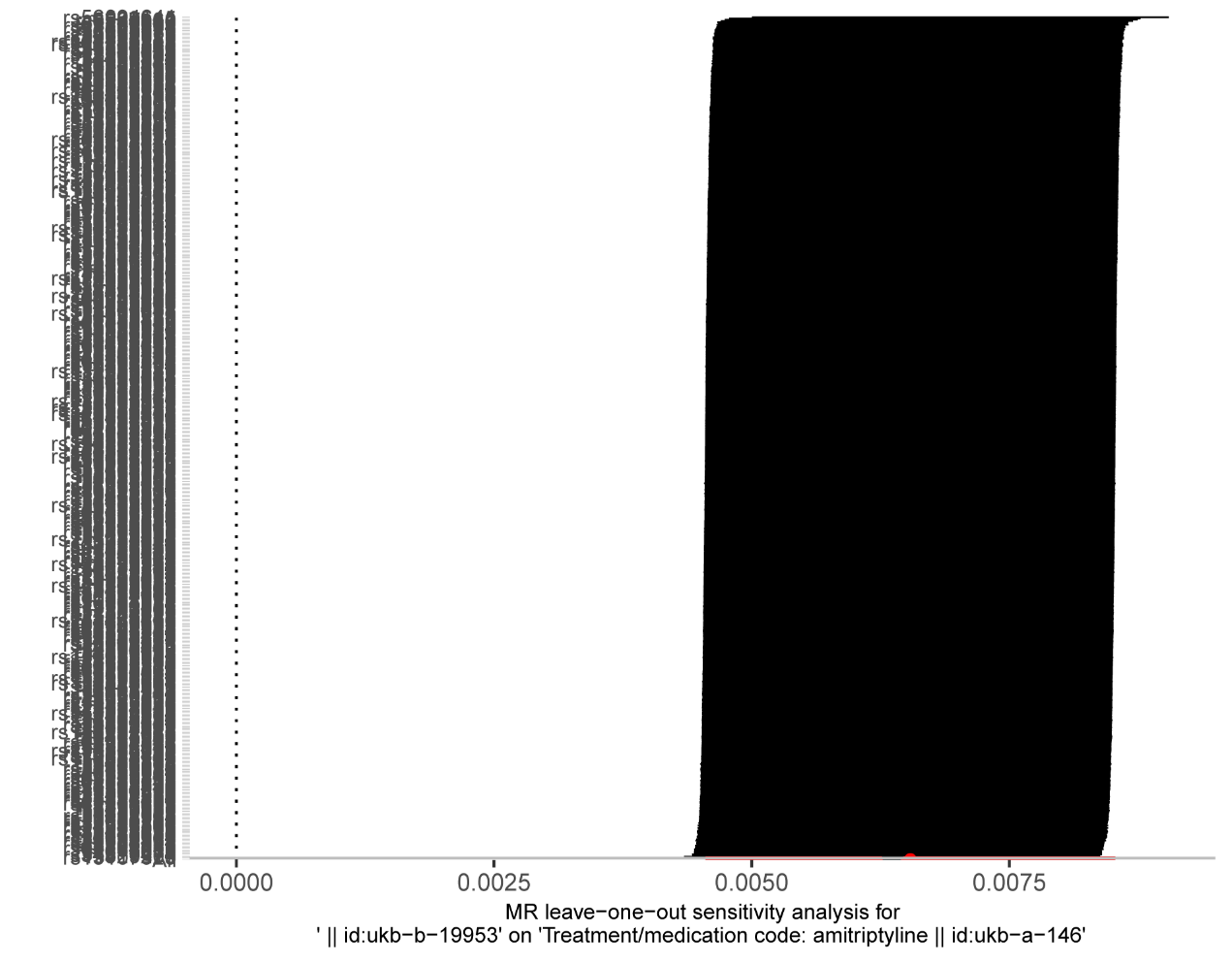


**Fig.S14 the leave-one-out plot of the effect of body mass index on amitriptyline**

Supplement: Supplementary file 18 [file medi-103-e38668-s018.docx]

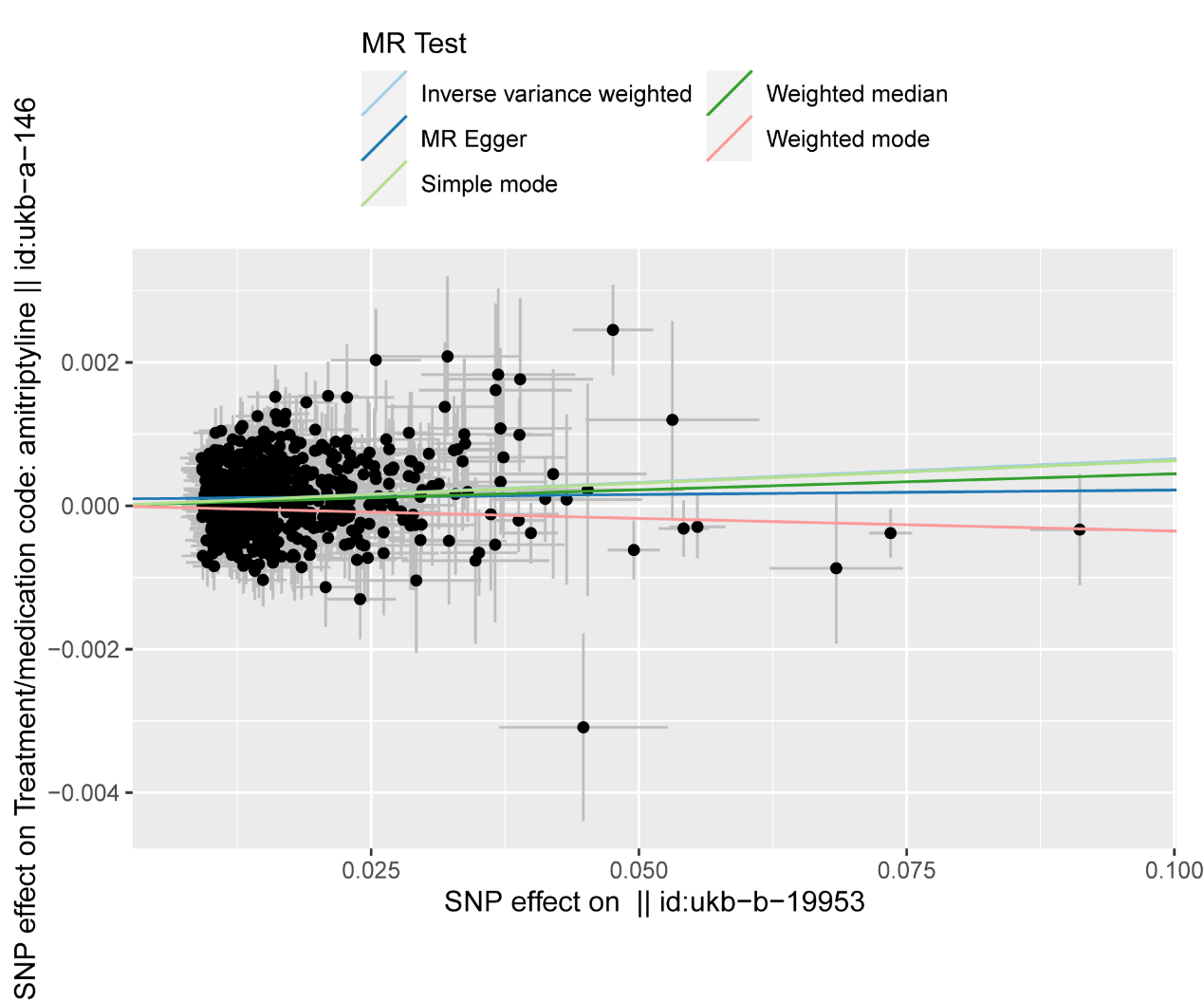


**Fig.S15 the scatter plot of the effect of body mass index on amitriptyline**

Supplement: Supplementary file 19 [file medi-103-e38668-s019.docx]

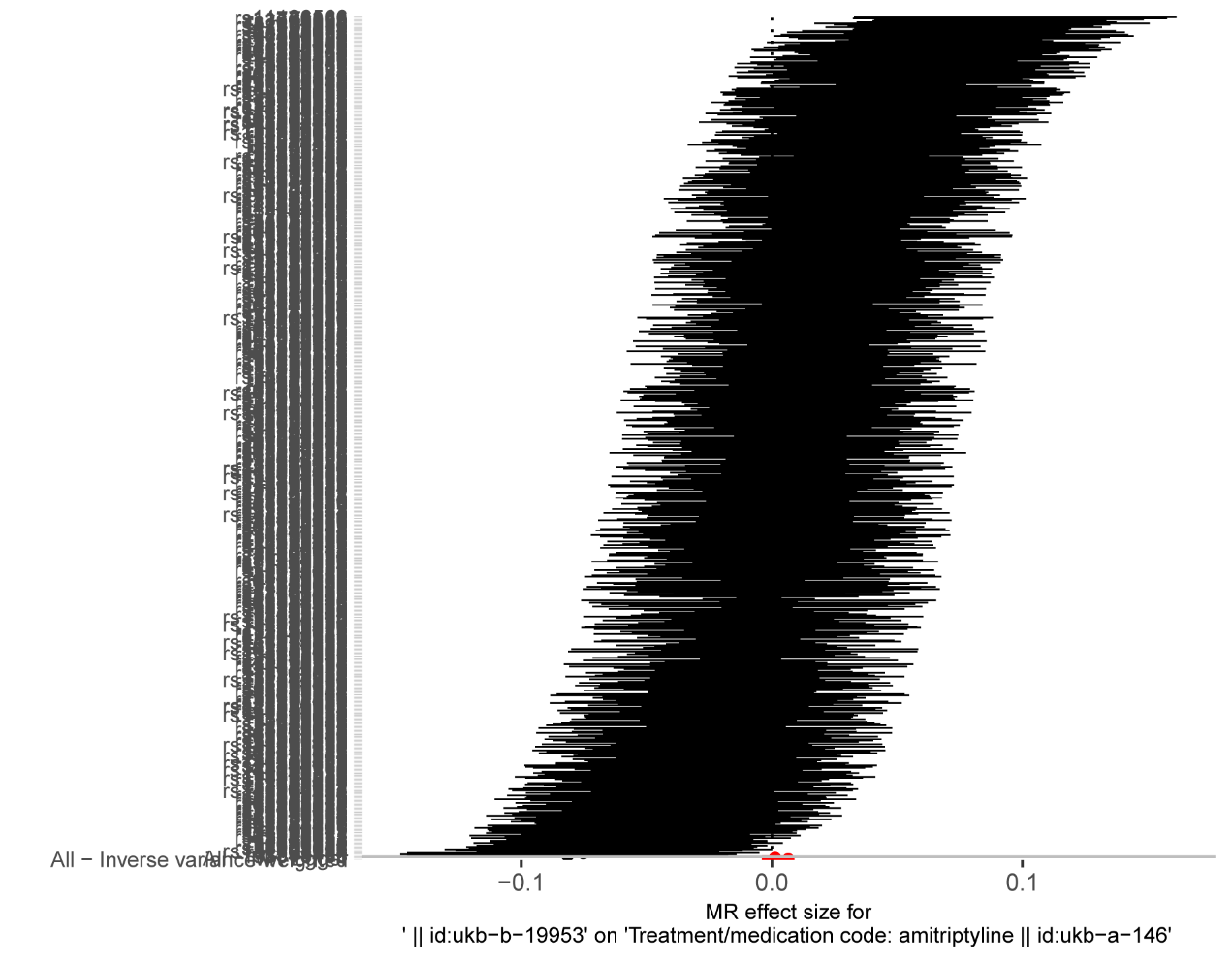


**Fig.S16 the forest plot of the effect of body mass index on amitriptyline**

Supplement: Supplementary file 20 [file medi-103-e38668-s020.docx]
