# Supplementary material for: Effectiveness and safety of Chaihu-Shugan-San for treating depression based on clinical cases: An updated systematic review and meta-analysis
Source: Medicine (Baltimore). 2024 Jun 28;103(26):e38668. doi: 10.1097/MD.0000000000038668 (PMC11466128; doi:10.1097/MD.0000000000038668)
Supplement: Supplementary file 3 [file medi-103-e38668-s003.docx]

**
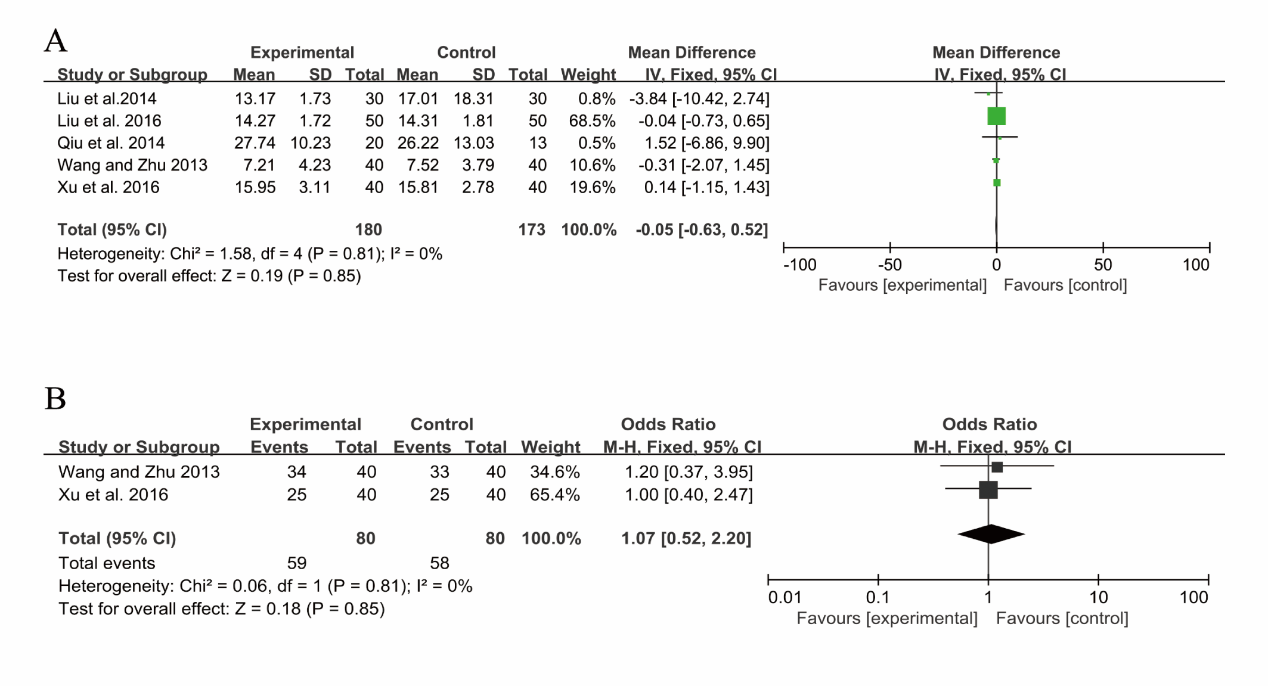
**

**Fig. S2 Outcome measurements after treatment: Chaihu-Shugan-San versus antidepressants alone.**

Note: A: The effect of CSS vs. antidepressants on the Hamilton Depression Rating Scale scores (HAMD)

B: The effect of CSS vs. antidepressants on the effective rate
