# Supplementary material for: Effectiveness and safety of Chaihu-Shugan-San for treating depression based on clinical cases: An updated systematic review and meta-analysis
Source: Medicine (Baltimore). 2024 Jun 28;103(26):e38668. doi: 10.1097/MD.0000000000038668 (PMC11466128; doi:10.1097/MD.0000000000038668)
Supplement: Supplementary file 5 [file medi-103-e38668-s005.docx]

| Table S1. Meta-Regression. | | | | | | |
| --- | --- | --- | --- | --- | --- | --- |
| _ES | Coefficient | Std. err. | t | P > \|t\| | [ 95% conf. interval ] | |
| year | 0.2579329 | 0.1456137 | 1.77 | 0.137 | -0.1163789 | 0.6322448 |
| Antidepressants | 0.4393764 | 0.3598348 | 1.77 | 0.276 | -0.4856086 | 1.364361 |
| Length of intervention | -0.7789853 | 0.2876809 | -2.71 | 0.042 | -1.518493 | -0.039478 |
| _cons | -518.8193 | 291.7257 | -1.78 | 0.135 | -1268.724 | 231.0855 |
