# Supplementary material for: Effectiveness and safety of Chaihu-Shugan-San for treating depression based on clinical cases: An updated systematic review and meta-analysis
Source: Medicine (Baltimore). 2024 Jun 28;103(26):e38668. doi: 10.1097/MD.0000000000038668 (PMC11466128; doi:10.1097/MD.0000000000038668)
Supplement: Supplementary file 11 [file medi-103-e38668-s011.docx]

| **Table S3 Characteristics of SNPs used as genetic instruments for amitriptyline in the present MR study** | | | | | | | | | | |
| --- | --- | --- | --- | --- | --- | --- | --- | --- | --- | --- |
| SNP | Chr | Position | EA | OA | SNP-Amitriptyline | | | SNP-Body mass index (BMI) | | |
|  |  |  |  |  | Beta | SE | *P* | Beta | SE | *P* |
| rs115494124 | 10 | 65750299 | T | C | 0.004938 | 0.001064 | 3.51E-06 | 0.019005 | 0.006424 | 0.0031 |
| rs117502522 | 8 | 26497089 | G | C | 0.005503 | 0.001075 | 3.09E-07 | 0.015267 | 0.006428 | 0.018 |
| rs12988670 | 2 | 67539863 | G | A | -0.001702 | 0.000365 | 3.13E-06 | -0.004653 | 0.002166 | 0.032 |
| rs13105581 | 4 | 103228830 | T | C | 0.00296 | 0.000622 | 1.93E-06 | 0.034077 | 0.003692 | 2.70E-20 |
| rs13288990 | 9 | 9836873 | T | C | 0.002652 | 0.00058 | 4.82E-06 | -0.001778 | 0.003434 | 0.6 |
| rs1370895 | 3 | 165629972 | G | A | 0.001649 | 0.000348 | 2.17E-06 | 0.000502 | 0.002068 | 0.81 |
| rs148035962 | 4 | 60188540 | G | A | 0.008689 | 0.00186 | 2.98E-06 | -0.003156 | 0.011299 | 0.780001 |
| rs16863064 | 3 | 187887577 | C | G | 0.002222 | 0.000473 | 2.63E-06 | -0.001018 | 0.002773 | 0.709999 |
| rs17163983 | 5 | 128024582 | A | T | -0.002038 | 0.000404 | 4.61E-07 | -0.002044 | 0.002409 | 0.4 |
| rs2289582 | 15 | 75310394 | C | T | -0.002134 | 0.000426 | 5.55E-07 | -0.005758 | 0.002515 | 0.022 |
| rs2655705 | 13 | 61550237 | C | T | 0.002089 | 0.000438 | 1.87E-06 | 0.003335 | 0.00261 | 0.2 |
| rs28724242 | 6 | 32629331 | G | A | 0.001834 | 0.000391 | 2.68E-06 | 0.005564 | 0.00232 | 0.016 |
| rs2901785 | 1 | 174104743 | A | G | -0.001635 | 0.000334 | 1.00E-06 | 0.00143 | 0.001981 | 0.47 |
| rs4147355 | 18 | 50478364 | A | G | 0.00172 | 0.000333 | 2.37E-07 | 0.006468 | 0.001985 | 0.0011 |
| rs4819521 | 22 | 19765322 | T | C | -0.001544 | 0.000338 | 4.76E-06 | -0.004325 | 0.002012 | 0.032 |
| rs568150 | 1 | 229872298 | C | T | 0.00242 | 0.000494 | 9.84E-07 | 0.00192 | 0.002949 | 0.52 |
| rs62436128 | 6 | 153341774 | T | C | 0.005717 | 0.001207 | 2.19E-06 | 0.002923 | 0.007094 | 0.68 |
| rs76539580 | 1 | 104803191 | T | C | -0.00582 | 0.001241 | 2.73E-06 | -0.009557 | 0.007463 | 0.2 |
| rs77910031 | 10 | 72792424 | A | G | 0.005814 | 0.001209 | 1.50E-06 | 0.000281 | 0.007166 | 0.97 |
| rs79977266 | 4 | 172480598 | G | A | 0.005958 | 0.001226 | 1.18E-06 | -0.016286 | 0.007381 | 0.027 |
| rs9913079 | 17 | 47519174 | C | T | 0.001791 | 0.000379 | 2.32E-06 | 0.005904 | 0.002257 | 0.0089 |
| Abbreviation: SNP, single nucleotide polymorphism; MR, Mendelian randomization; Chr, chromosome; EA, effect allele; OA, other allele; SE, standard error.a The SNPs having a direct effect on the outcome (P < 5E-8) were remove from the primary MR analysis. | | | | | | | | | | |
