# Supplementary material for: Effectiveness and safety of Chaihu-Shugan-San for treating depression based on clinical cases: An updated systematic review and meta-analysis
Source: Medicine (Baltimore). 2024 Jun 28;103(26):e38668. doi: 10.1097/MD.0000000000038668 (PMC11466128; doi:10.1097/MD.0000000000038668)
Supplement: Supplementary file 12 [file medi-103-e38668-s012.docx]

| Table S4. MR results of the causal effect of MR and reverse MR. | | | | | | | | | |
| --- | --- | --- | --- | --- | --- | --- | --- | --- | --- |
| Exposure | Outcome | N of SNPs | Method | OR (95%CI) | *P ^a^* | *beta* | Heterogeneity test | Pleiotropy test | |
|  |  |  |  |  |  |  | Cochran's Q | *P* Intercept | *P* Global Test |
| amitriptyline | BMI | 21 | IVW | 5.95 (1.77, 20.00) | 0.00393975 | 1.783421734 | 20 (2.02e-13) |  |  |
|  |  | 21 | WM | 2.63 (0.13, 55.03) | 0.54134197 | 0.965492454 |  |  |  |
|  |  | 21 | MR-Egger | 2.54 (1.10, 6.38) | 0.03022729 | 0.972797568 |  | 0.571 |  |
|  |  | 18 | MR-PRESSO (Outlier-corrected) |  |  |  |  |  | <0.001 |
| BMI | amitriptyline | 671 | IVW | 1.01 (1.00, 0.01) | 1.14E-10 | 0.006539455 | 669 (1.12e-4) |  |  |
|  |  | 671 | WM | 1.00 (1.00, 1.01) | 0.63749685 | 0.001251491 | 670 (7.24e-5) |  |  |
|  |  | 671 | MR-Egger | 1.00 (1.00, 0.01) | 0.00366616 | 0.00446177 |  | 0.032 |  |
|  |  |  | MR-PRESSO (Outlier-corrected) |  |  |  |  |  | <0.001 |
| Abbreviation: MR, Mendelian randomization; SNPs, single nucleotide polymorphisms; OR, odds ratio; CI, confidence interval; IVW, inverse variance weighted; WM, weighted median; MR-PRESSO, MR-pleiotropy residual sum and outlier. ^a^ To account for multiple testing, we used Bonferroni-corrected thresholds of 0.005 (α = 0.05/10) in our MR analyses. We considered *P* below the threshold as significant evidence of associations | | | | | | | | | |
